# Children's interactions with iPad books: research chapters still to be written

**DOI:** 10.3389/fpsyg.2013.00995

**Published:** 2013-12-24

**Authors:** Natalia Kucirkova

**Affiliations:** Department of Education, The Open UniversityMilton Keynes, UK

**Keywords:** iPads, early literacy, shared book reading, new literacies, digital books

Children's interactive e-books are novel literacy tools with interactive and multimodal representations of story contents and increased customizable features. The learning opportunities represented by these new affordances demand a thorough consideration of children's engagement, including the contextual and socio-cultural factors which influence the books' deployment in home and classroom settings. Currently, there is inconclusive evidence about how the affordances of interactive e-books support children's learning, with studies mostly limited to comparison studies with non-digital books and observational studies of children's immediate engagement. In both lines of research, the content of the stories, the overall context of interaction and the background of the interactants are neglected. This article makes recommendations for future research and highlights the value of iPads as a new medium enriching children's experiences but also challenging traditional research assumptions.

## Children's iPad books: affordances and distinctive features

Children's interactive e-books are touch-manipulative digital books which represent meanings in three modes (i.e., sound, text, and image) and offer several possibilities for interaction (e.g., main character moves when touched). They differ from paper-based books and from classic PC books according to several dimensions, summarized in Table1.

**Table 1:** **Overview of affordances of three main reading tools for children**.

	**Audio representation**	**Visual representation**	**Touch screen**	**Interactivity**	**Customization**	**Personalization**
Books	No	Yes	No	No	No	No
PC books	Yes	Yes	No	Yes	Yes	Yes
E-books^*^	Yes	Yes	Yes	Yes	Yes	Yes

* E-books can be downloaded to various devices, including smartphones, Kindle and iPads.

Children's interactive e-books can be downloaded on several devices such as smartphones and tables. Those downloaded on iPads can be of two kinds: those created by the users themselves using any of the “book-making apps” available in App store (e.g., My Story™, Our Story) or “iPad storybooks” which are ready-made and can be downloaded from the ibook store (e.g., Cinderella™, What is That?). In practice, book-making apps often accompany iPad storybooks or are even integrated into them (e.g., the Farmer's Lunch app by HarperCollins). In this piece, I focus on interactive e-books downloaded on iPads and subsume under the term “iPad books” books created with book-making iPad apps or those which are commercially available.

iPad books come with several activities (e.g., possibility for coloring the storybook pages) and customization or personalization options. The features which have not been available for children's books before are increased interactivity, multimodal story representation and unprecedented customizability of reading experience. Considering these novel features and the increased popularity and growing industry of children's iPad books (Costello, 2012), it seems important to research the books' potential for supporting children's learning and engagement with literacy.

## Research to date: issues and promising avenues

So far, research concerning children's iPad storybooks has focused on two approaches: observational studies nested in qualitative research and comparison studies which tend to adopt quantitative research design. In the former approach, researchers have documented children's engagement with a variety of iPad apps, including book-making (or story-making) apps in classrooms. For example, Hutchison et al. (2012) investigated how iPads support children's reading and responding to text in elementary classrooms. Several apps were investigated, including three book-making apps: iBooks, Strip Designer and Popplet. Teachers' reports and the researchers' observations indicated that iBooks were primarily used for independent reading in the classroom, while the Strip Designer and Popplet apps were used for children's creative story-composing. Both kinds of books seemed to engage and motivate children to practice new literacy skills, such as cooperation, creativity or self-revision. Similarly, Flewitt et al. (2012) have documented young children's engagement with various apps in a primary and pre-school classroom, including a book-making app called Our Story. Children were found to be meaningfully and creatively engaged with Our Story and the teachers positively commented on the app's potential to support children's digital literacy and inventiveness.

In another line of research, Parish-Morris et al. (2013) compared children's touch-sensitive electronic console books (comparable to iPad storybooks) and traditional books in relation to dialogic language and children's story comprehension. The authors observed 165 parent–child dyads and found that children's story comprehension was negatively influenced by the presence of electronic features in the electronic console books. Chiong et al. (2012) investigated the differences between basic e-books (i.e., digital books with minimal interactive and customization features) and enhanced e-books, as read by 32 parents and their 3–6-year-old children. Chiong et al. analyzed their observational data along several dimensions, including physical and verbal engagement, story recall, as well as verbal labeling, or pointing in relation to specific story features. The findings showed that the enhanced interactivity of e-books negatively affected children's story recall but was advantageous for engaging children in the activity and prompting physical interaction.

Thus, it seems to be the case that in comparison studies, iPad books fare less well than traditional books, but when studied in their own right, iPad books are reported to engage children and to have positive effects different from simple digital books. However, in drawing these conclusions, it is important to recognize some persistent issues in research concerned with children's interactions with technology. Moreover, methodological inconsistences among the studies limit the insights into the research implications. This leaves many practitioners uncertain about how to best support children's interaction with the tools (Kucirkova, 2013). To help guide future research and development in this area, I suggest some theoretical and methodological considerations.

## Theoretical pointers

I argue that to empirically illustrate the novel affordances of iPad books, researchers need to follow theories which view digital texts as a new medium of communication, and new technologies as tools which afford novel learning spaces. Researchers who evaluate children's learning experiences need to consider traditional as well as new digital literacy skills which are opening out with new technologies. For the latter, children's ability to interpret digital images or to collaborate on a piece of text are relevant (Wolsey and Grisham, 2012). Such an approach resonates with the proponents of the New Literacies Framework, who associate new technologies with new definitions of literacy (Coiro et al., 2008). New Literacies Framework puts forward the idea that new technologies reshape the ways in which stories are presented and interpreted (Lankshear and Knobel, 2003) and that their mastering and understanding requires new literacy skills. Such a position builds upon previous theoretical work which views new forms of books as new literacy tools with unique affordances for meaning-making (Salomon, 1992). These views are supported by historical evidence outlined by Madej (2003) who reviewed the evolution of interactive features in digital texts and concluded that new digital texts are more than an extension or replacement of previous books. Even though there were various media accompanying books as early as in the 1980s (such as an audio tape or television show to enrich a paper-based story), there was a considerable shift of story presentation in the 1990s, with new technologies “reshaping stories” (Madej, 2003, p.15). Therefore, researchers interested in finding out how novel features of iPad books affect children's learning, need to adopt a theoretical framework which facilitates conceptualizing iPad books as unique, 21st century learning tools. This is closely linked to the need for adopting a detailed and innovative methodology to ascertain the books' value for children's learning.

## Methodological pointers

Some steps in adopting new methodological approaches to study children's iPad books have been already undertaken. For example, Falloon (2013) applied an innovative methodology to generate and analyse data from the use of iPads by eighteen 5-year-olds in a New Zealand preschool classroom. Faloon used the Display Recorder app with which he could record children's interaction from within the device, generating video files with detailed finger placement indication and good sound quality. This procedure allowed the researcher to collect data reflecting children's natural interaction and minimize observer-effect which often occurs from close observation or video recording. Moreover, a complex list of factors influencing children's engagement was obtained, including effects which reflect teachers' ability to adequately support the children, such as modeling the child's response or offering corrective and formative feedback (Falloon, 2013). Although a new analytical framework might pose difficulties for direct comparisons with findings from previous research with traditional paper-based books, it offers powerful opportunities for future design of iPad books, both in terms of their content and format features. It is likely that a thorough consideration of the nature of interactive features, designed with or without a pedagogic intention in mind, will help designers gather specific data on the process of children's engagement and establish possible areas of concern.

Existent qualitative methodologies offer another promising research avenue in this respect. Kucirkova et al. (2013) looked in detail at a parent-child interaction when reading a self-made book on iPad. A detailed multimodal interactional analysis approach allowed the authors to analyse the interaction along verbal and non-verbal interaction indices (Norris, 2004) and to distinguish between verbal and non-verbal communication and “embodied (gesture, gaze and language) and disembodied (e.g., a book, iPad, and picture) resources used for meaning-making” (Kucirkova et al., 2013, p. 117). In addition to the analysis of communicative modes of the parent and child, Kucirkova et al. considered the communication modes of the audio-visual book, i.e., the sounds and images which were part of the story the mother and child viewed and interacted with during story-sharing. The study showed that parent-child sharing of the personal iPad book can give rise to a new interactional space, resembling the atmosphere of experiencing a piece of art. The study thus underscored the importance of future research to consider the possibility that iPad books can establish a new interaction context which needs to be studied with comprehensive and detailed methodologies.

In addition to methodological innovations and creative research approaches, future research agendas need to combine innovation with some well-known, yet often neglected, issues in technology research. In this respect, my recommendations for future research focus on three areas.

First, future research needs to consider children's engagement in light of factors related to interaction with technology. Notably, children's previous exposure to iPads, technological competency, gender and overall literacy skills are likely to play a role in children's engagement with iPad books (see Plowman, 1995).

Second, future research should involve an examination of the interlinked relationship between iPads' use and home and classroom literacy practices. It has been well established that the use of books is heavily influenced by the classroom practice preceding their introduction (Ertmer, 1999; Windschitl and Sahl, 2002) and that teachers' beliefs and instructional philosophy are often a more powerful determinant of technology use than the technology itself (Miller and Olson, 1994). It follows that for books presented in the form of digital apps or texts, teachers' classroom practice and their conception of the nature of children's interaction with books and technology are vital variables to be included in analyses. By the same token, parents' attitudes and beliefs about appropriate early literacy activities ought to be considered as a crucial element in future parent-child-iPad book investigations (see e.g., Bingham, 2007).

Finally, future research should study in detail the *content* of the iPad storybooks children engage with. In light of the well-established effects of different book contents on children's learning (e.g., Nyhout and O'Neill, 2013), it is important to enrich existing findings with a detailed examination of the variety of contents iPad books can accommodate, ranging from self-generated innovative storybooks through conventional alphabet and information books.

In conclusion, iPad books for children are a novel addition to the rich repertoire of children's books in the 21st century. A small but emerging literature exists which shows inconclusive evidence of the books' benefits for young children. In view of the current belief that new literacy activities ought to foster children's current *and* future literacy skills (Barone and Mallette, 2013), researchers need to be mindful of issues related to the evaluation of new and traditional literacy practices. Future research needs theoretically sound and methodologically innovative examinations of the ways in which iPad books add to children's literacy experiences.
